# Seasonal variation and correlation analysis of vitamin D and parathyroid hormone in Hangzhou, Southeast China

**DOI:** 10.1111/jcmm.15330

**Published:** 2020-05-16

**Authors:** Miaoda Shen, Zhuoyang Li, Duo Lv, Ge Yang, Ronghuan Wu, Jun Pan, Shuo Wang, Yifan Li, Sanzhong Xu

**Affiliations:** ^1^ Department of Orthopedics The First Affiliated Hospital College of Medicine Zhejiang University Hangzhou China; ^2^ Research Center of Clinical Pharmacy The First Affiliated Hospital College of Medicine Zhejiang University Hangzhou China; ^3^ Department of Orthopedics Hunan Children's Hospital Changsha China

**Keywords:** 25‐hydroxyvitamin D, parathyroid hormone, seasonal variation, Southeast China, vitamin D

## Abstract

This study aimed to describe the 25‐hydroxyvitamin D (25(OH)D) and parathyroid hormone (PTH) status of Southeast Chinese individuals influenced by season. The secondary aim was to determine the cutoff for sufficient 25(OH)D in a four‐season region. From January 2011 to June 2014, a total of 17 646 individuals were evaluated in our study. The serum levels of PTH were detected simultaneously in 5579 cases. A total of 25(OH)D and intact PTH were measured by the electrochemiluminescent immunoassay. The distribution of the concentration, prevalence and seasonal variability of 25(OH)D and PTH were studied. The mean 25(OH)D concentration in our study was 43.00(30.40) nmol/L. The prevalence of insufficiency (25(OH)D < 50 nmol/L) was 62.87% and that of deficiency (<30 nmol/L) was 28.54%. Mean serum 25(OH)D levels revealed a limited sinusoidal profile throughout the year and were significantly higher in Autumn. On the other hand, PTH levels showed an opposite response to seasonal effects relative to 25(OH)D. Age, BMI and daylight were not significantly correlated with 25(OH)D and serum PTH reached a plateau at higher values of serum 25(OH)D of 42.86 nmol/L. This study demonstrated that Vitamin D insufficiency is highly prevalent in Southeast China. The concentration of 25(OH)D in the male group was generally higher than that in the female group. Seasonal variation was an important aspect of 25(OH)D and PTH concentration. This study revealed that the optimal serum threshold of 25(OH)D for bone health should be between 40 and 50 nmol/L for Southeast Chinese individuals.

## 1 INTRODUCTION

Vitamin D deficiency is associated with unfavourable skeletal outcomes, including fractures and bone loss.^1^ Cholecalciferol (Vitamin D3) is the only type that can be synthesized inside the human body, which is produced in the skin from 7‐dehydrocholesterol with ultraviolet (UV) light. It is then transported to the liver bound to vitamin D‐binding protein (DBP) and metabolized to 25‐hydroxyvitamin D (25(OH)D), through an enzymatic process involving 25‐hydroxylase. Within the renal tubular cell, 25(OH)D undergoes 1‐alpha‐hydroxylase and 24‐alpha hydroxylase to form 1,25 (OH) 2, the active form of the vitamin.^2^ The National Osteoporosis Society (NOS) recommends that the serum concentration of 25(OH)D is the best indicator of Vitamin D status in the human body.^3^ And NOS consider a serum 25(OH)D threshold of 30 nmol/L for deficiency and potential for deficiency between 30 and 50 nmol/L.^4^ However, there is no universal consensus on the level of serum 25(O)HD which constitutes sufficiency and the appropriate thresholds for vitamin D deficiency are debated. A low vitamin D status is emerging as a very common condition worldwide. A recent study has suggested that 34% of Africans are vitamin D insufficiency (25(OH)D < 50 nmol/L), and 18.46% are deficient (25(OH)D < 30 nmol/L).^5^ Meanwhile, prevalence rates of vitamin D insufficiency, defined as 25(OH)D < 50 nmol/L, of 24% (US),^6^ 37% (Canada),^7^ 40% (Europe)^8^ and 58.7% (Korea)^9^ have been reported. Several reports on the vitamin D status in Chinese populations come from Taiwan,^10^ Southern^11^ and northwest^12^ China populations, but there are few studies on the current status of vitamin D in Southeast Chinese individuals, who have a different diet, lifestyle and climate.

From a clinical point of view, several factors may affect the production of vitamin D in the skin, including seasonal variation, gender, age, BMI and genetic factor.^13^, ^14^, ^15^ On the other hand, low Vitamin D level often leads to an increase of parathyroid hormone (PTH) and calcium release from bone. The secondary hyperparathyroidism will cause osteoporosis and pathological fractures. Recently, there has been considerable evidence that secondary hyperparathyroidism has developed for a long time before Vitamin D level reduces to deficiency. Therefore, it is suggested that the deficiency of Vitamin D should be defined as the lowest Vitamin D level that causes secondary hyperparathyroidism. As previously reported, this threshold, however, is not permanent and varies from 50 to 104 nmol/mL with different geographic location, country and season.^15^, ^16^, ^17^, ^18^ To our knowledge, there is no relevant study in Southeast China.

The present study aims to evaluate the serum 25(OH)D status among populations in Southeast China. Moreover, we analyse the changes in 25(OH)D and PTH levels by season and gender based on retrospective data. Further, we explore the relationship between the concentrations of serum 25(OH)D and PTH. Finally, we proposed a threshold that a minimum 25(OH)D serum concentration of 42.86 nmol/L was required to stabilize PTH levels.

## 2 MATERIALS AND METHODS

### 2.1 Study population

This study included the results of 17 646 Chinese individuals in Hangzhou (northern latitude 30°) who had measurements of serum 25(OH)D at the First Affiliated Hospital of Zhejiang University between January 2011 and December 2015. The serum levels of PTH were measured simultaneously in 5579 individuals. The extreme values were excluded by the Generalized Extreme Studentized procedure, leaving data from 8598 female and 8281 male individuals aged between 13 and 111 years (62.2 ± 18.8 years) to be used in the analysis. Informed consent was obtained from all subjects. The study protocol has been approved by the local Medical Ethics Committee of the First Affiliated Hospital, College of Medicine, Zhejiang University, China.

### 2.2 Clinical and biochemical measurements

All the subjects completed a standard questionnaire on demographic characteristics (eg age and sex) and medical history. Then, completed anthropometrical measurements with the assistance of trained staff using standard protocols. BMI was calculated as weight in kilograms divided by height in meters squared (kg/m^2^). The exclusion criteria were as follows: (a) chronic liver and kidney disease, (b) poorly controlled metabolic disorders like diabetes, (c) hereditary or metabolic bone disease except for osteoporosis, (d) malignant tumour, (e) long‐term bedridden and (f) previous use of drugs that affect bone metabolism such as cortisol. Fasting blood samples were collected in the morning. Serum 25(OH)D concentration and intact PTH concentration were measured by the electrochemiluminescent immunoassay (ECLIA) in a Cobas e411 Elecsys 2010 analyzer (Hitachi‐Technologies Corp., Tokyo, Japan) according to the manufacturer's instructions. The test ranges of 25(OH)D and PTH were 7.50‐175 nmol/L and 1.20‐5000 pg/mL, respectively. The subjects were stratified into five groups according to their age: <50 years old, 50‐59 years old, 60‐69 years old, 70‐79 years old and > 80 years old. Based on several research articles and society recommendations,^3^, ^19^ we used the following groups: >75 nmol/L sufficient, 50‐75 nmol/L moderate insufficient, 30‐50 nmol/L insufficient, 20‐30 nmol/L deficiency and <20 nmol/L severe deficiency.

### 2.3 Statistical analysis

The baseline characteristics were presented using means with the standard deviations or medians with the interquartile ranges for continuous variables, and frequencies with percentages for categorical variables. Differences between the seasons were examined using the one‐way ANOVA test and Kruskal‐Wallis test for continuous and ranked variables, respectively. Differences between the 25(OH)D levels were examined using Kruskal‐Wallis and chi‐square test for ranked and nominal variables, respectively. The generalized linear model was fitted to explore the correlation between 25(OH)D and PTH adjusted for age, gender and seasons. The boxplot and bar chart were coded using R version 3.5.1 (R Core team). All other statistical analyses were performed using IBM SPSS Statistics (version 21.0). Throughout, we refer to statistical significance as a two‐sided *P* < 0.05.

## 3 RESULT

### 3.1 Subjects characteristics

The participants’ demographic and biochemical information was provided in Tables 1 and 2. The mean age of all participants was 62.2 ± 18.8 years old (range from 13 to 111 years old). The proportion of males was lower than that of females: 46.4% vs 53.6%. The average BMI was 25.1 ± 4.0 kg/m^2^, of which 2535 cases were obese (15%). Among the biochemical parameters, the median value of 25(OH)D of the total was 43.00(30.40) nmol/L, of which 44.08 (31.83) nmol/L for males and 38.28 (23.80) nmol/L for females. The serum PTH concentration median was 49.80 (40.05) pg/mL. Furthermore, as shown in Table 3, we correlated 25(OH)D with other relevant parameters and found that PTH was the strongest negative correlation parameter for 25(OH)D (*P* < 0.001), while season was the strongest positive correlation parameter (*P* < 0.001), and there was a significant difference between autumn and spring (*P* < 0.001). In the remaining parameters, gender was significantly correlated with 25(OH)D (*P* < 0.001). Age, BMI and daylight were not significantly correlated with 25(OH)D (*P* = 0.592, 0.496 and 0.947, respectively).

**TABLE 1:** Overall baseline characteristics of participants

Variable	Spring (n = 1402)	Summer (n = 1455)	Autumn (n = 1436)	Winter (n = 1286)	All months (N = 5579)	*P*‐value
PTH (pg/mL)	80.27 ± 33.08	48.13 ± 23.78	40.11 ± 16.84	62.55 ± 31.84	57.47 ± 31.08	<0.001*
	81.40 (57.52)	47.60 (32.43)	36.95 (19.47)	56.70 (46.05)	49.80 (40.05)	
BMI (kg/m^2^)	25.1 ± 4.1	25.2 ± 4.0	25.1 ± 4.1	25.1 ± 4.0	25.1 ± 4.0	0.803*
25(OH)D (nmol/L)	40.81 ± 21.58	46.75 ± 24.32	52.37 ± 24.65	43.67 ± 22.62	45.99 ± 23.75	<0.001^#^
	37.50 (26.10)	44.00 (29.90)	50.61 (32.31)	40.44 (29.50)	43.00 (30.40)	
≤20 (n, %)	210 (15.0)	162 (11.1)	124 (8.6)	163 (12.7)	659 (11.8)	
20‐30 (n, %)	260 (18.5)	213 (14.6)	142 (9.9)	221 (17.2)	836 (15.0)	
30‐50 (n, %)	551 (39.3)	516 (35.5)	440 (30.6)	478 (37.2)	1985 (35.6)	
50‐75 (n, %)	277 (19.8)	407 (28.0)	491 (34.2)	309 (24.0)	1484 (26.6)	
>75 (n, %)	104 (7.4)	157 (10.8)	239 (16.6)	115 (8.9)	615 (11.0)	
Daylight (h)	12.74 ± 0.80	13.80 ± 0.86	11.62 ± 0.79	10.55 ± 0.39	12.18 ± 1.37	0.022*

Abbreviations: 25(OH)D, 25‐hydroxyvitamin D; BMI, Body mass index; PTH, parathyroid hormone.

* *P*‐value was calculated used the one‐way ANOVA test.

^#^ *P*‐value was calculated used the Kruskal‐Wallis test.

**TABLE 2:** Subgroup analysis by demographic characteristics according to 25(OH)D levels

	25(OH) D						*P* values
	≤20, n = 659	20‐30, n = 836	30‐50, n = 1985	50‐75, n = 1484	>75, n = 615	Total, N = 5579	
Gender, n (%)							
Male	304 (46.1)	380 (45.5)	877 (44.3)	706 (47.4)	322 (52.4)	2589 (46.4)	**<0.01** *
Female	355 (53.9)	455 (54.5)	1108 (55.7)	778 (52.6)	293 (47.6)	2990 (53.6)	
BMI, kg/m^2^	25.3 ± 4	25.3 ± 4	25.1 ± 4.1	25.1 ± 4.1	25.1 ± 4.0	25.0 ± 4.0	0.598^#^
Obesity, n (%)							
BMI ≤ 28 kg/m^2^	451 (68.4)	592 (70.8)	1414 (71.2)	1055 (71.1)	452 (73.5)	3964 (71.1)	0.402*
BMI > 28 kg/m^2^	208 (31.6)	244 (29.2)	571 (28.8)	429 (28.9)	163 (26.5)	1615 (28.9)	
PTH, pg/mL	66.09 ± 35.86	65.73 ± 33.99	59.43 ± 31.10	50.98 ± 26.38	46.38 ± 24.71	57.47 ± 31.08	**<0.001** ^#^
	59.10 (50.84)	57.80 (47.47)	56.30 (43.10)	47.90 (37.50)	40.64 (27.90)	49.80 (40.05)	
PTH category, n (%)							
≤65 (pg/mL)	372 (56.5)	467 (55.9)	1293 (65.1)	1130 (76.1)	506 (82.3)	3768 (67.5)	**<0.001** *
>65 (pg/mL)	287 (43.5)	369 (44.1)	692 (34.9)	354 (23.9)	109 (17.7)	1811 (32.5)	

Abbreviations: 25(OH)D, 25‐hydroxyvitamin D; BMI, Body mass index; PTH, parathyroid hormone.

^#^ *P*‐value was calculated used the Kruskal‐Wallis test.

* *P*‐value was calculated used the chi‐squared test.

*P* < .05 is considered statistically significance.

**TABLE 3:** Correlation analysis between 25(OH)D and PTH adjusted for age, gender and seasons

	*β*	SE	*P* (95% CI)
Age	−0.009	0.017	0.592 (−0.042, 0.024)
Gender	−2.041	0.618	<0.001 (−3.252, −0.830)
Female vs male	−2.198	0.622	<0.001 (−3.417, −0.979)
Season (vs Spring)	0.557	0.618	0.050 (−0.011, 1.126)
Summer	1.883	0.941	0.045 (0.039, 3.727)
Autumn	6.554	0.983	<0.001 (4.627, 8.482)
Winter	0.649	0.916	0.479 (−1.148, 2.445)
PTH	−0.126	0.011	<0.001 (−0.149, −0.103)
BMI	−0.052	0.077	0.496 (−0.203, 0.098)
Daylight	−0.232	0.056	0.947 (−0.967, 0.956)

### 3.2 Prevalence of serum 25(OH)D in the evaluated population of Southeast China

**FIGURE 1:**
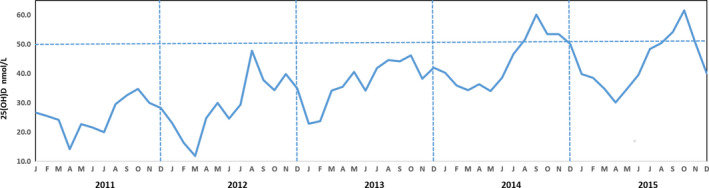
The changing trends of serum concentration of 25(OH)D by year and month

This study included 17 646 25(OH)D results from 2011 to 2015 were shown in Figure 1. The overall trend within one year was significantly higher in summer and autumn than in spring and winter. As mentioned above, vitamin D insufficient was considered to be present when 25(OH)D < 50 nmol/L, and 25(OH)D < 30 nmol/L indicated deficiency. Total vitamin D insufficiency rate was 62.4%, and the deficiency rate was 26.8% from 2011 to 2015. Serum 25(OH)D was <20 nmol/L in 11.8% of the subjects and >75 nmol/L in 11% of the study participants (Table 1, *P* < 0.001). Nevertheless, only a few months met the criteria of sufficiency in 2014 and 2015. Most of the time, the 25(OH)D level of Hangzhou citizen was in a state of insufficiency. Over time, however, the 25(OH)D increased almost every year compared with the previous (Figure 1).

### 3.3 Seasonal distribution of serum 25(OH)D and PTH concentrations in Southeast China population

**FIGURE 2:**
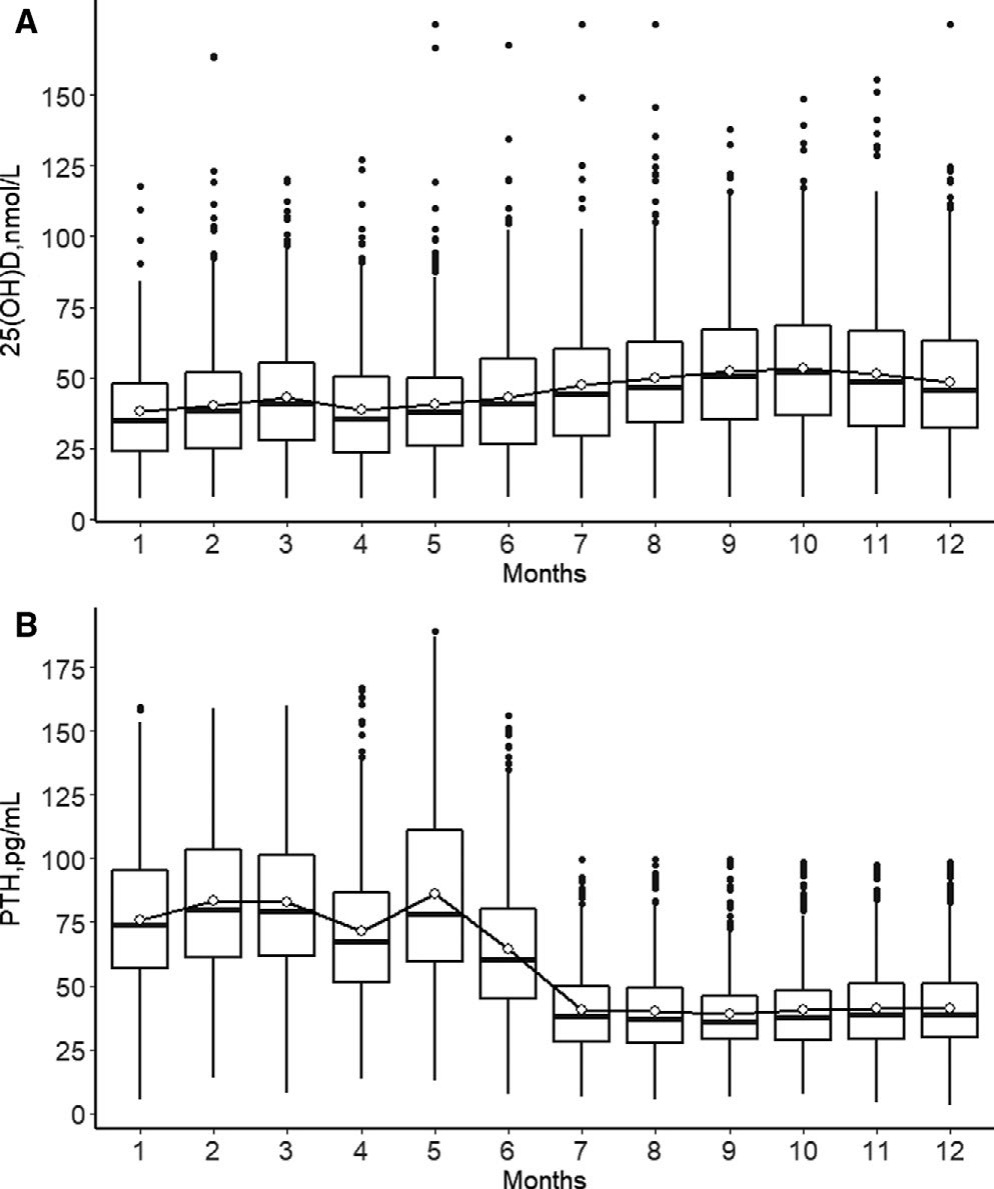
Changes in serum concentration of 25(OH)D (A) and PTH levels (B) by month

**FIGURE 3:**
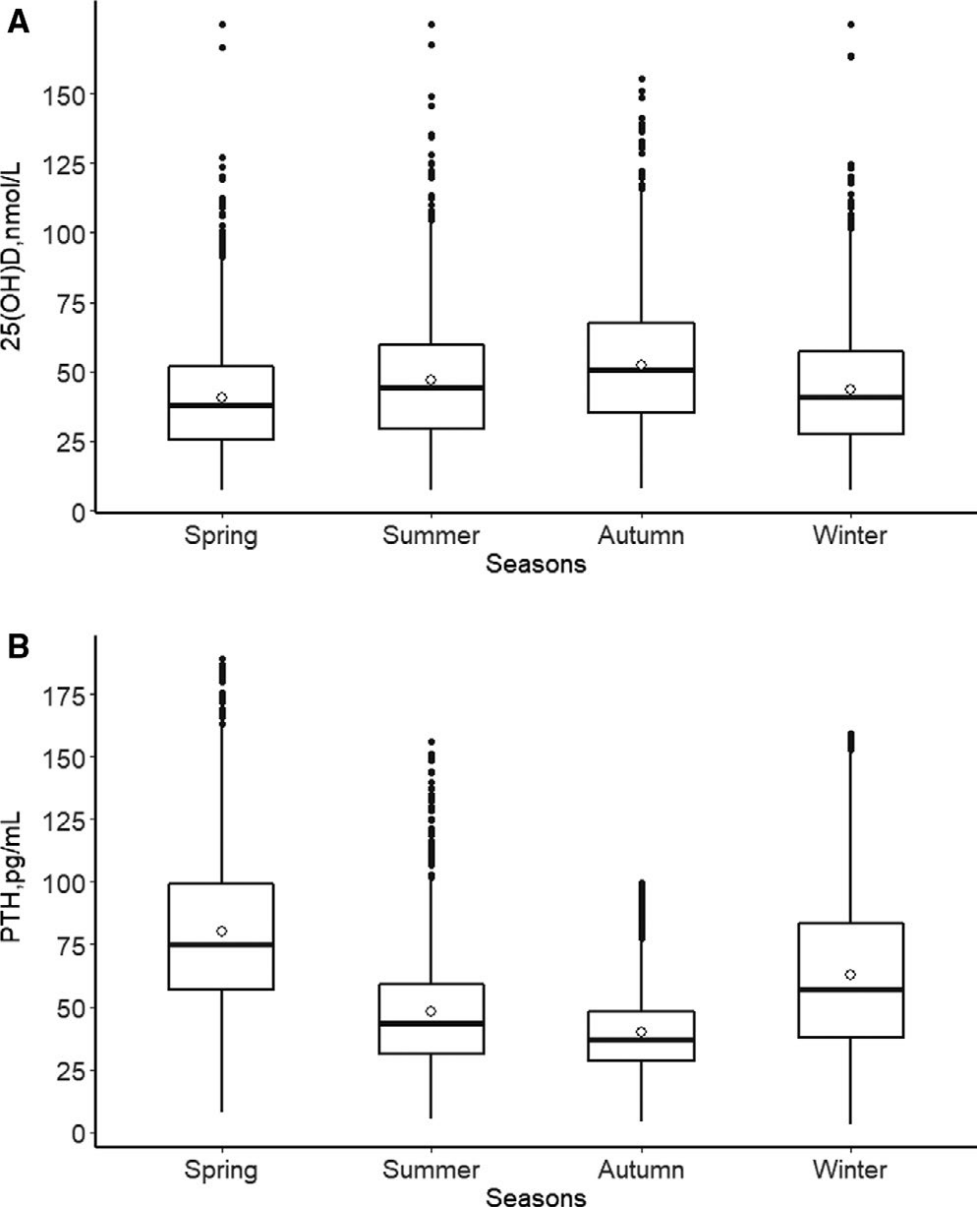
Seasonal variation of mean total serum 25(OH)D (A) and PTH (B) concentration

Both 25(OH)D and PTH showed a seasonal variation, the season‐specific classification of 25(OH)D results according to the functional groups is presented in Figures 2 and 3. The maximum seasonal variation of 25(OH)D (peak to trough) was 15 nmol/mL (Figure 3). Analysis of seasonal changes in 25(OH)D and PTH levels revealed a limited sinusoidal profile where the 25(OH)D values increased starting in April, reaching a peak level in October, and then decreased to baseline levels by January (Figure 2A). Meanwhile, seasonal changes in PTH levels also showed a limited inverse sinusoidal pattern (Figure 2B) to what was observed for 25(OH)D levels.

**FIGURE 4:**
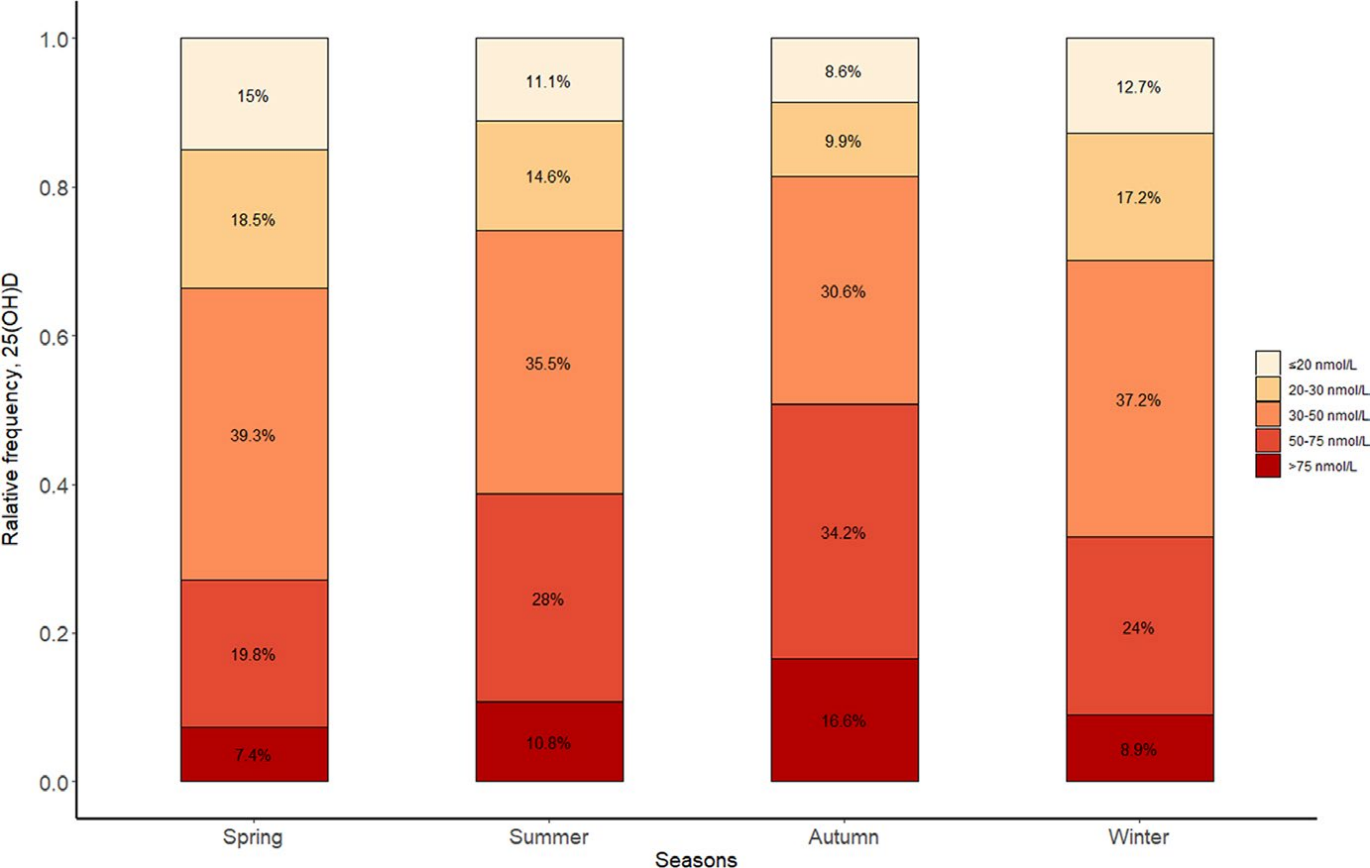
Relative deficiency frequency of serum 25(OH)D according to the stratified groups and seasons

It should be noted that more than 90% of people had 25(OH)D lower than 75 nmol/L between November and May. The percentage of people with insufficiency 25(OH)D (<50 nmol/L) levels dropped to 49.1% during the autumn months (severe deficiency in 8.6%) (Figure 4 and Table 2, *P* < 0.001). In all groups, regardless of any season, the highest proportion was always in the insufficiency group (average 35.6%, ranging from 30.6% to 39.3%). The sufficiency group was always the lowest, average 11.0%, range from 7.4% to 16.6% (Figure 4, *P* < 0.001). Consistent with this, participants with a high PTH level (PTH > 65 pg/mL) accounted for 32.5% of the total population (Table 2, *P* < 0.001).

### 3.4 Determinants of 25（OH）D level and association with PTH the correlation between concentrations of serum 25(OH)D and PTH

**FIGURE 5:**
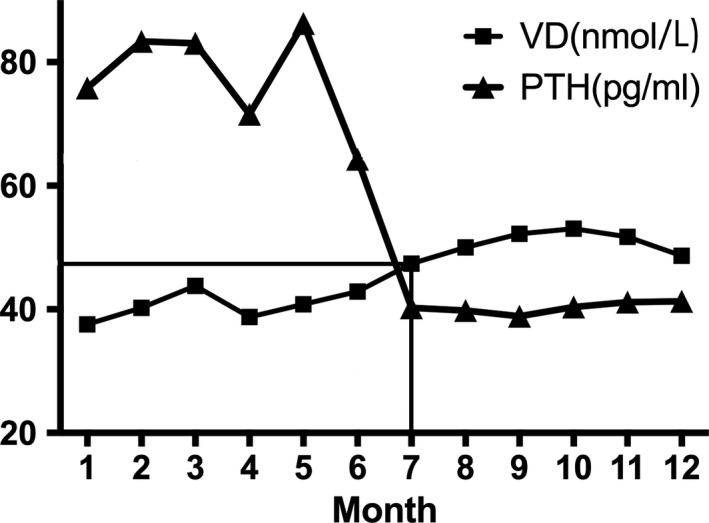
The relation between PTH and 25(OH)D levels. The lines indicated that when 25(OH)D > 42.86 nmol/L (July), PTH began stable in the range of 35‐40 pg/mL

The subgroup analysis of 25(OH)D found that the concentration of 25(OH)D in the male group was generally higher than that in the female group (Table 2, *P* < 0.01). There was no significant difference in 25(OH)D between obese and normal BMI participants across subgroups (*P* > 0.05). The model indicates an inverse significant correlation between PTH and 25(OH)D levels. Figure 5 showed that when the 25(OH)D increased, the corresponding proportion of high PTH subjects decreased (from 43.5% to 17.7%). On the contrary, the PTH level reached its highest value in spring 81.40 (57.52) pg/mL and lowest in autumn 36.95 (19.47) pg/mL. The changes in PTH were significantly different across the seasons (*P* < 0.001). The average concentration of PTH was in the normal range in summer and autumn.

Lastly, we compared the monthly 25(OH)D and PTH in all groups and found the cutoff in July (Figure 5). Before July, there was a negative correlation between the concentrations of 25(OH)D and serum PTH. We observed that the PTH level showed a steeper decrease when 25(OH)D level was up to 50 nmol/L in July. And serum PTH reached a plateau at higher values of serum 25(OH)D of 42.86 nmol/L.

## 4 DISCUSSION

This study of nearly 17 646 25(OH)D and 5579 PTH test results represent the largest aggregation of data used to illustrate the 25(OH)D status, the prevalence of vitamin D deficiency and the correlation between serum concentrations of 25(OH)D and PTH in a Southeast China population. To the best of our knowledge, this is the first study to focus on a large sample of individuals from Southeast China across the entire age range and overall seasons in a long‐span period.

The general 25(OH)D concentration in males was higher than that in females, and other studies also reported consistent results, especially in the Middle East and Asia.^11^, ^15^ This may be associated with the higher dietary intake of vitamin D and longer sun exposure in males.

Although we did not find a significant difference between BMI and 25(OH) D, the mean values showed that 25(OH)D was slightly lower in obese participants (Table 2). Vitamin D is fat‐soluble and distributed into fat, muscle, liver and serum. All of these compartments are increased in volume in obesity, so the whole body stores of vitamin D may be adequate.^20^, ^21^

Another unexpected finding from this study revealed that the daylight time does not correlate with the serum 25(OH)D level. The daylight time reached the highest at the end of June (14 hours 7 minutes) in Hangzhou according to the weather data, while the highest serum 25(OH)D level presented in autumn. The lag time between vitamin D and daylight peak may relate to an accumulative effect of sunlight exposure over the summer months and the relatively long half‐life of circulating 25 (OH)D. On the other hand, 25 (OH)D does not reach their lowest values until at least two months following the winter solstice, again reflecting the cumulative effect of decreased or absent cutaneous vitamin D synthesis during the winter months.^13^ Actually, sunlight exposure is just one of the factors which influence vitamin D status.^22^

25(OH)D deficiency has previously been detected at different rates depending upon geographical location and country. For example, Kroll et al^13^ reported a 35% average deficiency in the United States, while Cashman et al^8^ stated the deficient prevalence was 40.4% in Europe. According to the National Osteoporosis Society and the Endocrine Society guidelines,^3^, ^4^, ^23^ vitamin D deficiency is defined as a level of 25(OH)D lower than 30 nmol/L in our study. One strength of this study is the largest data set ever collected in the region which experiences four seasons. The percentage of subjects considered vitamin D deficient broadly ranged from a trough of approximately 18.5% in autumn to 33.5% in spring (Figure 3), 26.8% overall. According to an alternate suggested definition of vitamin D deficiency (<50 nmol/L), the prevalence was 62.4%. Our findings are consistent with the previous study of vitamin D deficiency in China showed a rather high deficiency rate, up to 55.9%.^24^ On the other hand, however, the 25(OH)D increased almost every year compared with the previous (Figure 1). This could be the results of vitamin D supplementation has increased during the last years in our country,^25^, ^26^ and accordingly, the prior situation of a high prevalence of vitamin D deficiency has improved in China.

Analysis of seasonal changes in 25(OH)D and PTH hormone levels revealed a sinusoidal pattern where the 25(OH)D values increased starting in April, reaching a peak level in October, and then decreased to baseline levels by January (Figures 1 and 2). We have shown that both 25(OH)D and PTH demonstrate a consistent inversely proportional seasonal sinusoidal pattern, and the peaks and troughs lag behind the times of the greatest or least amounts of daylight. Seasonal variations in serum 25(OH)D concentrations have been demonstrated for different populations at different latitudes.^27^, ^28^, ^29^ These studies guide the interpretation of results based on the season and current guidelines may need to consider the seasonality. Therefore, the results highlight the influence of seasonal variation on the adjustment of vitamin D supplementation.

25(OH)D deficiency can lead to an increase in serum PTH, which can cause bone resorption, osteoporosis and fractures. Our study indicated that there was a negative correlation between serum 25(OH)D and PTH levels (Figure 5). In recent years, more and more studies have demonstrated that some calcium and bone metabolism‐related changes, including a small amount of calcium absorption, bone loss and mild increases in PTH level, have occurred before 25(OH)D reaches clinical insufficiency.^30^ Many research groups try to establish the required serum 25(OH)D concentrations by assessing the threshold of 25(OH)D, below which serum PTH starts to increase. However, varying optimal serum levels of 25(OH)D were recommended to be used as clinical thresholds of the deficiency by different research groups. For example, Olmos et al^16^ suggested a threshold of serum 25(OH) D level of 75 nmol/L would be necessary for the prevention of secondary hyperparathyroidism and hip bone loss. Zhang et al^17^ reported the most optimal 25(OH)D levels to be between 30 and 50 nmol/L based on PTH hormone levels which tended to show a steep increase above optimal 25(OH)D levels chosen. In our analysis, PTH levels reach a stable plateau above 25(OH)D levels of 42.86 nmol/L suggesting this value to be the clinical decision threshold for 25(OH)D. These results indicate that the optimal serum threshold of 25(OH)D for bone health should be between 40 and 50 nmol/L.

The present study has some limitations and one of them is the lack of information on vitamin supplementation usage of the participates whose test results were used. The second one is that we measured serum 25(OH)D and PTH in a single clinical centre and bone turnover markers were not measured. A further study based on subjects sampled on multicenter of the different regions would be carried out and more biochemical parameters, such as bone density and bone turnover markers should be investigated. Nevertheless, the present study is valuable because it was based on retrospective data employed from the largest study group ever in the Southeast China population which experiences four seasons.

## 5 CONCLUSION

In conclusion, our study demonstrates that vitamin D insufficiency is highly prevalent in Southeast China. Total vitamin D insufficiency rate was 62.4%, and the deficiency rate was 26.8% from 2011 to 2015. The implication for our study population is that at least 62.4% should receive vitamin D supplements because their serum 25(OH)D concentrations are <50 nmol/L. This study confirms previous reports demonstrating a seasonal, inverse relationship between 25(OH)D and PTH. We suggest that each centre may need to consider seasonal changes to make the clinical diagnosis. Lastly, based on our analysis, at least 42.86 nmol/L of 25(OH)D is needed to achieve suppressed PTH levels. Bone health in the participants is likely to improve when serum 25(OH)D is raised to concentrations of 30‐50 nmol/L.

## CONFLICT OF INTEREST

The authors have no conflicts of interest to declare.

## AUTHOR CONTRIBUTIONS

Sanzhong Xu designed the research study; Miaoda Shen and Zhuoyang Li performed the research; Jun Pan, Shuo Wang and Yifan Li collected data; Dou Lv, Ge Yang and Ronghuan Wu analysed the data; Sanzhong Xu, Miaoda Shen and Zhuoyang Li wrote the paper.

## Data Availability

The data that support the findings of this study are available on request from the corresponding author. The data are not publicly available because of privacy or ethical restrictions.
